# How can brain imaging reshape our understanding of chronic pain?

**DOI:** 10.1192/j.eurpsy.2026.10339

**Published:** 2026-07-31

**Authors:** C. Siopa, F. Novais, D. Telles-Correia

**Affiliations:** 1 ULS Santa Maria; 2 Unidade Local de Saude de Santa Maria; 3 Faculty of Medicine, University of Lisbon, Lisbon, Portugal

## Abstract

**Background:**

Chronic pain is a complex and multidimensional experience that persists beyond normal tissue healing and affects a substantial proportion of the adult population. Traditional diagnostic frameworks have been strongly based on subjective reports, limiting the understanding of the neurobiological mechanisms underlying their onset, maintenance, and cognitive-emotional impact.

**Objective:**

This presentation aims to synthesize recent neuroimaging evidence elucidating how advances in brain imaging technologies are redefining our understanding of chronic pain, with implications for diagnosis, mechanistic models and personalized therapies.

**Methods:**

We reviewed contemporary findings from structural and functional neuroimaging studies - primarily magnetic resonance imaging (MRI), resting-state fMRI, and advanced connectivity analyses - as well as integrative meta-analyses and systematic reviews (PubMed up to 2025).

**Results:**

Emerging neuroimaging research demonstrates that chronic pain is associated with widespread brain alterations in both structure and function. Key findings indicate widespread structural and functional brain alterations in chronic pain. Structurally, individuals with chronic pain show consistent reductions in grey matter volume and disruptions in white matter integrity across regions including the insula, cingulate cortex, thalamus, hippocampus, motor areas, and cerebellum, resembling patterns associated with accelerated brain ageing and neurodegeneration. Functionally, altered resting-state connectivity within salience and default mode networks has been observed, along with variable yet meaningful changes in pain-related activation. Emerging evidence from machine learning approaches and direct neural recordings suggests the presence of pain-specific brain signatures with potential as objective biomarkers. Together, these neuroimaging findings provide mechanistic insight into disrupted pain modulation, affective–sensory integration, and large-scale network dysregulation, supporting the view of chronic pain as a disorder of the central nervous system rather than solely a peripheral condition.

**Discussion:**

Brain imaging has reshaped paradigms of chronic pain by moving beyond stimulus-response models toward network-based conceptualizations that integrate sensory, emotional and cognitive components. These methods challenge traditional clinical frameworks by offering more objective markers of pain experience and suggest potential targets for neuromodulatory and personalized interventions.

**Keywords:**

chronic pain, neuroimaging, MRI, functional connectivity, biomarkers, brain networks, chronic pain mechanisms.

**Disclosure of Interest:**

None Declared

